# Supramaximal Stimulus Intensity as a Diagnostic Tool in Chronic Demyelinating Neuropathy

**DOI:** 10.1155/2016/6796270

**Published:** 2016-06-16

**Authors:** Vivien Parker, Jodi Warman Chardon, Julie Mills, Claire Goldsmith, Pierre R. Bourque

**Affiliations:** ^1^Division of Neurology, University of Ottawa, Ottawa, ON, Canada K1Y 4E9; ^2^Ottawa Hospital Research Institute, Ottawa, ON, Canada K1Y 4E9; ^3^Department of Genetics, Children's Hospital of Eastern Ontario, Ottawa, ON, Canada K1H 8L1; ^4^Diagnostic Imaging, The Ottawa Hospital, Ottawa, ON, Canada K1Y 4E9

## Abstract

*Objective.* The ability to correctly identify chronic demyelinating neuropathy can have important therapeutic and prognostic significance. The stimulus intensity value required to obtain a supramaximal compound muscle action potential amplitude is a commonly acquired data point that has not been formally assessed as a diagnostic tool in routine nerve conduction studies to identify chronic neuropathies. We postulated that this value was significantly elevated in chronic demyelinating neuropathy.* Methods.* We retrospectively reviewed electrophysiology laboratory records to compare the stimulus intensity values recorded during median and ulnar motor nerve conduction studies. The groups studied included normal controls (*n* = 42) and the following diagnostic categories: chronic inflammatory demyelinating neuropathy (CIDP) (*n* = 20), acquired inflammatory demyelinating neuropathy (AIDP) (*n* = 13), Charcot Marie Tooth (CMT) type 1 or 4C (*n* = 15), carpal tunnel syndrome (CTS) (*n* = 11), and amyotrophic lateral sclerosis (ALS) (*n* = 18).* Results.* Supramaximal intensities were significantly higher in patients with CMT (median nerve: 43.4 mA) and CIDP (median nerve: 38.9 mA), whereas values similar to normal controls (median nerve: 25.3 mA) were obtained in ALS, CTS, and AIDP.* Conclusions.* Supramaximal stimulus intensity may be used as an additional criterion to identify the pathophysiology of neuropathy. We postulate that endoneurial hypertrophic changes may increase electrical impedance and thus the threshold of excitation at nodes of Ranvier.

## 1. Introduction

The ability of an electrical stimulus to excite the nerve depends on several factors, including electrical impedance of intervening tissues and axonal excitability. The stimulus traverses skin and soft tissues to reach the nodes of Ranvier then modifies axonal resting potential and activates sodium channels to generate an action potential. Transmembrane ion kinetics as well as nodal and paranodal properties determine how well the signal is generated and propagated. Additional factors that affect excitability are the impedance and capacitance of the paranodal space and perineural ensheathments.

We observed that a substantially greater stimulus intensity was commonly required to achieve a supramaximal compound action potential amplitude in patients with chronic demyelinating neuropathy. This observation had not been validated in the literature and or published in commonly used monographs on nerve conduction study techniques. Studies have examined axonal excitability in demyelinating and axonal neuropathies using threshold electrotonus, threshold tracking, refractoriness, supernormality, and strength-duration behavior [1, 2]. These techniques are not typically performed during routine nerve conduction studies for clinical purposes. We postulated that the supramaximal stimulus intensity, a value which is routinely acquired during conventional nerve conduction studies, may also provide information about nerve excitability to facilitate diagnosis and prognosis for patients with chronic demyelinating neuropathies. We studied the supramaximal stimulus intensities in normal subjects, patients with Charcot Marie Tooth (CMT), chronic inflammatory demyelinating polyneuropathy (CIDP), acute inflammatory demyelinating polyneuropathy (AIDP), and amyotrophic lateral sclerosis (ALS).

## 2. Methods

This was a retrospective study at a single center at The Ottawa Hospital. The study was approved by the Research Ethics Board.

### 2.1. Patients and Controls

A convenience sample was obtained using a database from the Neuromuscular Clinic to generate a list of patients with a diagnosis of CMTCIDP, AIDP, ALS, and CTS. The charts and electrodiagnostic studies from 2010 to 2015 were reviewed to achieve preset criteria.

Patients with demyelinating CMT were included if they had genetic confirmation of CMT1A or CMT4C. If genetic confirmation was not available, they had to have a clear documented family history, plus clinical and electrodiagnostic features deemed consistent with demyelinating CMT. No patients with CMTX were included. Axonal forms of CMT were excluded.

Patients with CIDP were included if they fit the INCAT criteria [3] and had evidence of involvement in the median or ulnar nerve, respectively (conduction velocity <40 m/s or distal motor latency >5.5 ms for median or >4.9 ms for ulnar nerve). Patients with AIDP were included if they fulfilled the Asbury criteria for AIDP [4]. Other variants such as acute motor axonal neuropathy (AMAN), acute motor and sensory axonal neuropathy (AMSAN), and Miller-Fisher syndrome were excluded.

Patients with ALS group were included if they fulfilled the revised El Escorial criteria for definite or probable ALS [5] or LMN syndrome in >3 spinal segments (not due to other causes), clinical involvement of the upper extremity tested and evidence of moderate axonal loss with amplitude reduction in compound muscle action potential (CMAP) between 1 and 6 mV.

Patients with CTS were included if they had evidence of demyelination without significant axonal loss in the median nerve studies based on preset electrodiagnostic criteria of distal motor latency >4.5 ms, distal sensory latency >3.5 ms, and no loss of amplitude in CMAP (>10 mV). The CTS group was compared with the normal controls to determine if there was any impact on stimulation intensities, as we could not control for incidental carpal tunnel syndrome in the CMT, CIDP, AIDP, and ALS groups.

Data from a normative study on healthy control subjects were retrospectively reviewed. Exclusion criteria for control subjects included a diagnosis of neuropathy, CTS, ulnar neuropathy, foot drop, sciatica, radiculopathy, GBS, CIDP, diabetes mellitus, prior or ongoing chemotherapy, more than 3 alcoholic drinks a day, weight greater than 250 pounds, leg swelling, numbness or paresthesias in the extremities, rheumatoid arthritis, heavily calloused hands, diagnosis of hepatitis C, HIV, Lyme, or vitamin B12 deficiency.

### 2.2. Nerve Conduction Studies and Supramaximal Stimulation Intensity Measurements

All electrophysiologic studies were performed on XLTEK Xcalibur EMG equipment (Natus Medical, California). Standard disposable self-adhesive 1 cm electrodes were used for all skin surface recordings and were applied to the muscle belly (G1) and adjacent tendon (G2). The same hand-held bipolar stimulator probe was used on all patients. Data were extracted for median and ulnar motor nerve conduction studies including distal motor latency, CMAP amplitudes, and conduction velocity. For the median nerve, stimulus was applied at the wrist and then elbow and recorded over the abductor pollicis brevis. For the ulnar nerve, stimulus was applied at the wrist and then below the elbow and recorded over the adductor digiti minimi.

Supramaximal stimulation intensities in milliamperes (mA) were routinely documented during nerve conduction studies. The stimulus intensity was increased in 5–10 mA increments to a level 10% higher than the point where the resultant waveform did not increase in amplitude or area.

### 2.3. Statistical Analysis

Supramaximal stimulation intensity (mA) obtained from patients with CMT, CIDP, AIDP, and ALS and healthy controls was compared using one-way ANOVA on SPSS (Version 20, 2011). Stimulation intensities for the median and ulnar motor nerve conduction studies were compared separately. Subsequently, we performed a separate analysis of the stimulation intensity for the median nerve in patients with CTS compared to healthy controls using the independent samples *t*-test.

## 3. Results

**Figure 1:**
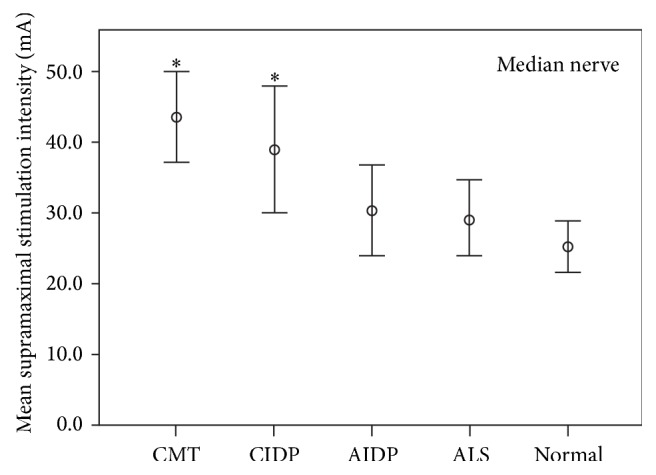
Mean supramaximal stimulus intensities (mA) from median motor nerve conduction studies and 95% confidence intervals for CMT, CIDP, AIDP, ALS, and normal controls. The median nerve was stimulated at the wrist. ^*∗*^Statistically significant (*p* < 0.05) higher mean supramaximal stimulus intensities in patients with CMT and CIDP compared to normal controls.

**Figure 2:**
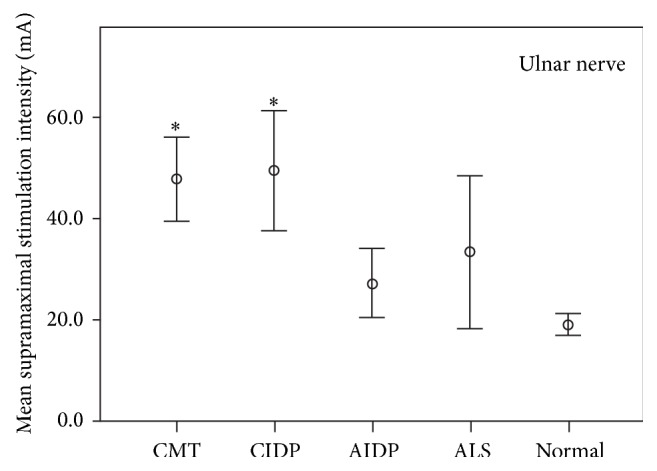
Mean supramaximal stimulus intensities (mA) from ulnar motor nerve conduction studies and 95% confidence intervals for CMT, CIDP, AIDP, ALS, and normal controls. The ulnar nerve was stimulated at the wrist. ^*∗*^Statistically significant (*p* < 0.05) higher mean supramaximal stimulus intensities in patients with CMT and CIDP compared to normal controls.

Supramaximal stimulus intensities were obtained from median and ulnar motor nerve conduction studies in normal subjects (*n* = 42 median and ulnar); CMT with demyelinating features (*n* = 15 median and ulnar); CIDP (*n* = 20 median, *n* = 16 ulnar); AIDP (*n* = 13 median and ulnar); and ALS (*n* = 18 median, *n* = 10 ulnar).  Figure 1 (median nerve) and Figure 2 (ulnar nerve) display supramaximal mean intensities and 95% confidence intervals for normal subjects and patients in the ALS, CMT, AIDP, and CIDP groups.

In median and ulnar nerve studies, mean supramaximal intensities were significantly higher in patients with CMT (median 43.4 mA; ulnar 47.7 mA) and CIDP (median 38.9 mA; ulnar 49.3 mA) than normal controls (median 25.3 mA; ulnar 19.0 mA) (*p* < 0.05). There was no significant difference in supramaximal intensities between CMT and CIDP. In the ulnar but not the median studies, higher supramaximal intensities were required in CIDP compared to AIDP (median 30.3 mA; ulnar 27.2 mA).

The AIDP patients did not differ significantly compared to controls for both the ulnar (*p* = 0.143) and median (*p* = 0.581) nerve studies. ALS patients had supramaximal intensities that did not differ significantly compared to the control population (ulnar nerve studies, *p* = 0.284; median nerve studies, *p* = 0.699).

Supramaximal stimulus intensities from the median motor nerve conduction studies were obtained for patients with carpal tunnel syndrome (CTS) meeting the preset criteria (*n* = 11). There was no significant difference in supramaximal intensities between patients with CTS and normal controls (26.9 mA and 25.3 mA, resp.).

## 4. Discussion

Supramaximal stimulus intensities measured with median and ulnar motor nerve conduction studies of patients with chronic demyelinating polyneuropathies, whether genetic (CMT) or acquired (CIDP), were significantly higher than normal controls. In contrast, ALS and AIDP patients had supramaximal intensity values similar to normal controls. This element of the routinely acquired electrophysiological testing data can thus provide a useful diagnostic clue to nerve pathophysiology.

With transcutaneous electrical stimulation of peripheral nerves, the electrical charge must first traverse the dermis and then subcutaneous extraneural tissues. There is an inverse relationship between impedance and current threshold, with greater impedance from fat or connective tissue compared to muscle [6]. Differences in subcutaneous tissue composition at the level of the wrist stimulation site are however not likely to have differentially affected the stimulation thresholds in the patient subgroups we studied, with the possible exception of adipose atrophy from malnutrition in ALS patients. This would be marginal at best at the level of the volar wrist, and our study did not reveal lower stimulation thresholds in ALS compared to control subjects.

Electrical charges must then traverse perineurium and endoneurium to reach nodes of Ranvier of individual motor axons. There are substantial endoneurial neuropathological alterations that could alter the flow of electrical charges in patients with CMT and CIDP and this may reflect the supramaximal stimulus intensities required in this study. Chronic dysmyelination in CMT and immune mediated demyelination in CIDP both lead to a pathologic hallmark of hypertrophic neuropathy. There is an increase in endoneurial collagen, with an increased number of layers of Schwann cell processes, as well as reduplication of basal lamina and the corresponding extracellular matrix proteins. In contrast, single wrapping of basal lamina is the main physical barrier found at the level of the normal node of Ranvier, where ionic currents generated by electrical stimulation mediate the change in axolemmal resting membrane potential that activates voltage-gated sodium channels. No significant perineurial changes have been documented in CMT, ALS, or CIDP.

The multiplication of basement membranes could also significantly increase electrical impedance. Transcellular electrical resistance measured by impedance spectroscopy demonstrates that basal membrane proteins laminin and collagen type IV have resistance values that are larger than rat tail collagen by a factor of 2.3–2.9 [7]. This may be particularly relevant to genetically determined hypertrophic neuropathies where onion bulb Schwann cell processes are more likely to disappear over time, leading to the formation of “basal lamina onion bulbs” [8].

In addition to reflecting electrical impedance of endoneurial tissues, the threshold for supramaximal stimulation is also a measure of nodal excitability. In CMT, immunostaining for contactin-associated protein spreads from paranodes to juxtaparanodes and internodes, while the voltage-gated potassium channels are redistributed from their normal juxtaparanodal localization [9]. Moreover, there can be remodeling of the nodal extracellular matrix in CMT type I, with displacement of tenascin from the node to an internodal location. The latter change could affect the ability to keep a reservoir of extracellular sodium ions in the perinodal space [10]. Node of Ranvier disruption is also important in immune mediated demyelinating neuropathies. In CIDP, contactin-associated proteins may be upregulated in the internodal axolemma and there is a decrease in Nav channel density at the level of the node [11].

Endoneurial edema and infiltration by inflammatory cells could also increase impedance, by producing physical barriers to the flow of current or by increasing the capacitance of the endoneurium, diverting charges away from the node of Ranvier. This mechanism is unlikely to have played a significant role in our study, as patients with AIDP had clearly lower supramaximal thresholds than the noninflammatory condition CMT.

Supramaximal stimulation thresholds of ALS patients did not differ from control subjects. Neuropathological studies in ALS show little endoneurial alterations other than axonal loss and a reduction in nerve cross-sectional area [12]. In addition, nodal Nav channel expression is maintained in ALS, whereas juxtaparanodal potassium channel immunoreactivity is lost in motor roots, a phenomenon that would correlate with axonal* hyperexcitability* [13].

Exacerbation of focal demyelination at common sites of entrapment would not likely confound results of supramaximal intensity determination in patients with AIDP, CIDP, or even CMT. Patients with median nerve entrapment at the level of the carpal tunnel may be expected to show conduction slowing, conduction block, and eventual axonal loss. However, transcutaneous electrical stimulation is performed over a more proximal portion of the median nerve, several centimeters proximal to the transverse carpal ligament. Nerve compression did not alter excitability measures when recorded 2 cm distally from a very localized compression point [14]. We found that supramaximal stimulation thresholds did not differ between patients with carpal tunnel syndrome and normal subjects.

A potential limitation of our study is that data was not obtained in a blinded prospective fashion but relied on a retrospective analysis of existing clinical records. This is not likely to have affected stimulation intensity values as the supramaximal motor potential amplitude was obtained with a standardized technique of gradual increments in stimulus intensity. ALS was selected as a prototypical example of axon-loss motor neuropathy. This approach is consistent with published literature on ALS [15] that is often quoted when analyzing the effect of axonal loss on nerve conduction study parameters such as motor conduction velocity and distal latency [16]. One should however be cautious in generalizing this inference to other forms of neuropathy (toxic or metabolic, e.g.) where axonal loss is generally thought to be the predominant pathophysiologic mechanism. The sample size was relatively small for the CMT and AIDP subgroups. Finally, this study did not assess supramaximal stimulus intensity in sensory conduction studies.

In conclusion, a significantly raised stimulus intensity required for supramaximal stimulation may provide a clue to the diagnosis of chronic demyelinating neuropathy. We postulate that increased electrical impedance from hypertrophic endoneurial changes is the predominant explanation.
